# 克唑替尼治疗洛拉替尼耐药伴*MET*扩增的*EML4-ALK*基因融合阳性晚期肺腺癌1例

**DOI:** 10.3779/j.issn.1009-3419.2024.102.37

**Published:** 2024-12-20

**Authors:** Xinyi WANG, Ning MU, Mei LIU, Yue XU, Shengnan WU, Huan LV, Chunhua MA

**Affiliations:** 300121 天津，天津市人民医院，南开大学第一附属医院肿瘤5科; Department of Oncology 5, Tianjin Union Medical Center, The First Affiliated Hospital of Nankai University, Tianjin 300121, China

**Keywords:** 肺肿瘤, ALK基因融合, MET扩增, 洛拉替尼, 克唑替尼, 分子靶向治疗, Lung neoplasms, ALK gene fusion, MET amplification, Lorlatinib, Crizotinib, Molecularly targeted therapy

## Abstract

肺癌是引起全球肿瘤死亡的重要原因，其中间变性淋巴瘤激酶（anaplastic lymphoma kinase, ALK）基因融合的患者占非小细胞肺癌（non-small cell lung cancer, NSCLC）的3%-7%。近年来，多种酪氨酸激酶抑制剂（tyrosine kinase inhibitors, TKIs）类药物显著改善了转移性ALK基因融合阳性NSCLC患者的生存情况，但仍有患者因耐药而发生疾病进展。本文回顾性分析1例棘皮类微管关联蛋白样4（echinoderm microtubule associated protein like 4, EML4）-ALK融合V3亚型的晚期肺腺癌患者在洛拉替尼治疗耐药后伴间质-上皮细胞转化因子（mesenchymal-epithelial transition factor, MET）扩增，应用克唑替尼与洛拉替尼联合靶向治疗后患者的肿瘤病灶有效控制，为临床进一步探索ALK-TKIs耐药后治疗模式提供参考。

## Abstract (en)

Lung cancer is a major cause of cancer-related mortality worldwide. Among patients with non-small cell lung cancer (NSCLC), approximately 3%-7% harbor anaplastic lymphoma kinase (ALK) gene fusions. In recent years, multiple tyrosine kinase inhibitors (TKIs) have significantly improved the survival of patients with metastatic ALK-positive NSCLC. However, disease progression due to resistance remains a challenge. This article retrospectively analyzes a case of advanced lung adenocarcinoma with the echinoderm microtubule associated protein like 4 (EML4)-ALK fusion variant 3 (V3). The patient developed resistance to Lorlatinib treatment accompanied by mesenchymal-epithelial transition factor (MET) amplification. Effective tumor control was achieved with the combined use of Crizotinib and Lorlatinib, providing a valuable reference for further exploration of treatment strategies following resistance to ALK-TKIs in clinical practice.

间质-上皮细胞转化因子（mesenchymal-epithelial transition factor, MET）基因融合在非小细胞肺癌（non-small cell lung cancer, NSCLC）中的发生率为3%-7%，其中棘皮类微管关联蛋白样4（echinoderm microtubule associated protein like 4, EML4）-间变性淋巴瘤激酶（anaplastic lymphoma kinase, ALK）是ALK基因融合NSCLC的主要变体，以EML4-ALK V1和V3亚型最为常见，占EML4-ALK变体总数的75%-80%^[1]^。研究^[2]^表明，EML4-ALK V3亚型或伴有TP53突变的ALK基因融合阳性NSCLC患者预后相对较差，而V3亚型伴TP53共突变的患者预后更差。本文报道1例EML4-ALK融合V3亚型伴TP53共突变的晚期肺腺癌患者经洛拉替尼（Lorlatinib）治疗16个月后，出现疾病进展（progressive disease, PD），胸水标本基因检测结果提示EML4-ALK融合V3亚型伴MET扩增，应用克唑替尼（Crizotinib）与洛拉替尼联合靶向治疗后患者的肿瘤病灶得到有效控制，现报道如下。

## 1 病例资料

患者，女性，71岁，2023年1月因“咳嗽伴喘憋，活动后加重1周”就诊于外院，行胸部电子计算机断层扫描（computed tomography, CT）检查，考虑左肺癌，予支持治疗后症状缓解。2023年2月因左侧颈部水肿于外院再行胸部CT检查，提示左肺恶性占位伴双侧肺内及多发淋巴结转移，合并左侧胸腔积液及心包积液，肿瘤原发灶-淋巴结-转移（tumor-node-metastasis, TNM）分期为T3N3M1a IV期。2023年2月6日行颈部淋巴结穿刺活检，病理结果提示：转移性低分化癌，倾向腺癌。同期穿刺组织标本送基因检测，结果回报为：EML4-ALK（6号内含子：19号内含子）重排；程序性细胞死亡配体1（programmed cell death ligand 1, PD-L1）：肿瘤细胞阳性比例分数（tumor proportion score, TPS）为85%，联合阳性分数（combined positive score, CPS）为90%，微卫星不稳定性（microsatellite instability, MSI）为6.04%，肿瘤突变负荷（tumor mutational burden, TMB）为5.01 Muts/Mb；TP53 c.853G>A p.E285K 8号外显子错义变异。2023年2月14日患者为行分子靶向治疗就诊于我院，开始洛拉替尼100 mg qd口服。肿瘤标志物（2023年2月18日）细胞角质蛋白19片段（cytokeratin-19 fragment, CYFRA21-1）：305 ng/mL。胸部增强CT（2023年2月22日）示：左下肺门软组织结节，符合肺癌表现；左肺下叶多发转移瘤，双侧锁骨上窝、颈后三角区、纵隔内、贲门旁多发淋巴结转移（图1A）。2023年9月23日患者入院复查，肿瘤标志物CYFRA21-1：0.209 ng/mL。胸部增强CT（2023年9月25日）示：左下肺门结节、左肺下叶微结节及双侧锁骨上窝、腋窝、颈后三角区、纵隔内、贲门旁多发淋巴结转移灶均较前减小（图1B）。肿瘤疗效评价为部分缓解（partial response, PR）。2024年2月19日患者再次入院复查，肿瘤标志物CYFRA21-1为3.21 ng/mL。胸部增强CT示（2024年2月20日）：左肺下叶微结节较前无著变，双侧锁骨上窝、腋窝、颈后三角区、纵隔内、贲门旁多发淋巴结转移，较前略增大，心包及左侧胸腔少量积液（图1C）。肿瘤疗效评价为疾病稳定（stable disease, SD），院外继续洛拉替尼100 mg qd口服分子靶向治疗。2024年6月29日患者因“喘憋伴咳嗽咳痰近1周”入院，肿瘤标志物CYFRA21-1：35.2 ng/mL，较前明显增高。胸部CT（2024年6月29日）示：相较于2024年2月20日检查结果，左肺下叶微结节此次显示不清，双侧锁骨上窝、纵隔内、贲门旁、腋窝多发淋巴结转移，左侧大量胸腔积液（图1D）。肿瘤疗效评价为PD。2024年7月1日行胸腔穿刺引流，同日予培美曲塞800 mg联合卡铂500 mg静脉化疗，2024年7月5日起予2次贝伐珠单抗联合顺铂胸腔灌注化疗，间断引流胸腔积液后患者喘憋症状较前好转，化疗后患者发生IV度骨髓抑制，体能状况（performance status, PS）评分为3分，予对症支持治疗。2024年7月16日患者再次出现喘憋，引流出大量乳糜状胸腔积液，送检基因检测及病理检查，2024年7月18日胸水病理结果回报：见核异型细胞，免疫组化考虑为肺腺癌；基因检测结果回报：ALK基因融合：EML4-ALK（E6:A20），MET基因拷贝数扩增，扩增倍数：4.0；MSI为微卫星稳定（microsatellite stability, MSS）。结合基因检测及病理结果，该患者为EML4-ALK V3亚型伴MET扩增的肺腺癌，2024年7月20日起口服单药克唑替尼靶向治疗，250 mg bid。复查床旁胸部X线提示胸腔积液较前明显减少（2024年7月16日，图2A；2024年7月23日，图2B），胸腔积液肿瘤标志物（CYFRA21-1）较前下降（2024年7月1日：405 ng/mL vs 2024年7月17日：10.3 ng/mL），考虑克唑替尼靶向治疗有效。2024年8月16日患者再入院，肿瘤标志物CYFRA21-1：34.1 ng/mL，胸部CT检查示：左侧胸腔积液较前减少，双侧锁骨上窝、腋窝、纵隔、贲门旁多发淋巴结转移灶部分增大（图2C）。头部增强磁共振成像（magnetic resonance imaging, MRI）（2024年8月19日）示：左侧小脑半球异常信号，转移瘤可能性大。疗效评价为脑部病灶局部进展，予调整靶向治疗用药，克唑替尼250 mg qd口服，联合洛拉替尼50 mg qd口服，后患者头晕、喘憋症状较前好转出院。患者2024年9月3日因神志恍惚就诊于我院，血细胞分析（2024年9月4日）示：白细胞计数：14.55×10^9^/L，中性粒细胞绝对值：12.34×10^9^/L，快速C反应蛋白：205.45 mg/L，肿瘤标志物CYFRA21-1：4.95 ng/mL，较前明显降低。2024年9月4日患者因与肿瘤进展无关的肺部感染导致呼吸衰竭去世。该患者克唑替尼与洛拉替尼联合治疗16 d，克唑替尼治疗洛拉替尼耐药后的无进展生存期（progression-free survival, PFS）为47 d。

**图 1:**
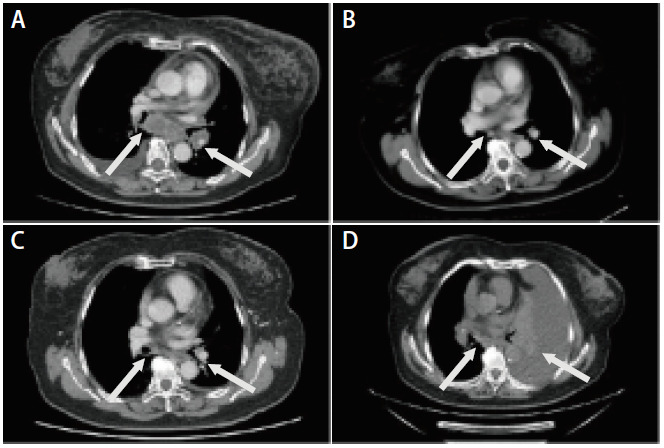
洛拉替尼单药治疗期间患者胸部CT特征。A：胸部CT纵隔窗（2023年2月22日）提示左下肺门软组织结节，直径约3.0 cm，符合肺癌表现，纵隔内及贲门旁等多发淋巴结转移；B：胸部CT纵隔窗（2023年9月25日）提示治疗后左下肺门软组织结节及纵隔内、贲门旁等多发淋巴结转移灶均较前明显减小；C：胸部CT纵隔窗（2024年2月20日）提示肺内病灶较前无著变，纵隔淋巴结等较前略增大；D：胸部CT纵隔窗（2024年6月29日）提示左侧大量胸腔积液。

**图 2:**
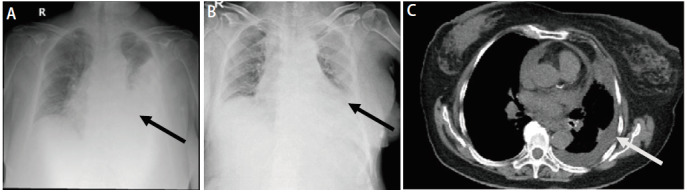
克唑替尼联合洛拉替尼治疗期间患者胸部影像特征。A：胸部正位X线（2024年7月16日）提示左侧大量胸腔积液；B：胸部正位X线（2024年7月23日）提示左侧积液量较前减少；C：胸部CT纵隔窗（2024年8月16日）提示左侧胸腔积液较前减少。

## 2 讨论

ALK基因是NSCLC中常见的致癌基因之一，通过与启动子基因（如EML4）的重排导致ALK融合蛋白的表达，这些ALK融合蛋白二聚化后激活ALK激酶，引起RAS-MAPK、PI3K-AKT-mTOR和JAK-STAT等下游信号通路转导失控，诱导肿瘤细胞增殖分化^[3]^。近年来，酪氨酸激酶抑制剂（tyrosine kinase inhibitors, TKIs）类药物在ALK融合阳性晚期NSCLC患者中展现出优异的治疗效果，目前国内已获批上市的药物包括第一代ALK-TKIs克唑替尼，第二代ALK-TKIs塞瑞替尼（Ceritinib）、阿来替尼（Alectinib）、布格替尼（Brigatinib）、恩沙替尼（Ensartinib）以及第三代ALK-TKIs洛拉替尼，极大地改善了ALK融合阳性转移性NSCLC患者的预后^[4]^。

本病例患者病理类型为肺腺癌，基因检测结果提示为EML4-ALK融合V3亚型伴TP53突变。一项全球II期研究结果^[5]^显示，洛拉替尼对既往接受过至少1次ALK-TKIs治疗的患者有效，客观缓解率（objective response rate, ORR）为47%，颅内反应率为63%，在既往接受过至少1次第二代ALK-TKIs治疗的患者中，ORR为40%，PFS为6.9个月。III 期CROWN研究^[6]^结果显示，洛拉替尼作为晚期ALK融合阳性NSCLC患者一线治疗，疗效明显优于克唑替尼，PD或死亡风险降低72%，中枢神经系统进展时间显著延长（96% vs 60%的患者在12个月时存活，无中枢神经系统进展；HR=0.07，95%CI: 0.03-0.17）。因洛拉替尼在ALK融合阳性NSCLC患者中良好的治疗效果，2024年美国国立综合癌症网络NSCLC指南将洛拉替尼推荐为晚期ALK融合阳性NSCLC患者治疗的一线治疗方案。

部分研究表明洛拉替尼的疗效会受到EML4变体类型的影响。III期CROWN研究^[7]^中比较了ALK-TKIs在EML4-ALK变异亚型间及是否伴随TP53突变或者其他潜在耐药突变的疗效，结果显示对于EML4-ALK融合V1、V2亚型，洛拉替尼组对比克唑替尼组的ORR更高（V1亚型：80.0% vs 50.0%；V2亚型：85.7% vs 50.0%；其他亚型：86.7% vs 66.7%），对于EML4-ALK V3亚型，无论是否合并TP53突变，洛拉替尼与克唑替尼相比均可改善ORR（73.9% vs 72.2%）和PFS（33.3 vs 5.5个月）。Zhang等^[2]^比较了6种上市ALK-TKIs对EML4-ALK融合V1和V3亚型的半数抑制浓度（half-maximal inhibitory concentration, IC_50_）。结果显示，在EML4-ALK融合V1亚型中，所有ALK-TKIs的IC_50_均不同的倍数低于V3亚型，提示了V3亚型对ALK-TKIs更具抗性，这可能是由于其内部具有更紧密的蛋白质构象从而增加了细胞内蛋白质的稳定性，引发了相对和统一的内在“抵抗”模式。因此，我们应该考虑在EML4-ALK融合V3亚型患者病程早期引入更积极的治疗。此外，TP53突变在多种肿瘤中均是常见的基因组改变，其可通过破坏P53蛋白肿瘤抑制功能促进肿瘤发展，在EML4-ALK融合变体中约有20%患者伴有TP53突变，这部分患者易发生更多的转移和更差的预后^[8]^。基于以上研究结果及指南推荐，我们在本例患者一线治疗中应用洛拉替尼100 mg qd口服，服药期间定期复查，肿瘤疗效评价为PR。患者服药16个月后再次出现胸闷憋气等症状，结合入院影像学表现疗效评价为PD。患者再行病理及基因检测（胸水），结果提示为EML4-ALK融合V3亚型伴MET扩增的肺腺癌。

近期有关洛拉替尼耐药机制的研究^[9,10]^表明，约1/3患者发生由ALK激酶结构域突变诱导的获得性耐药，而2/3患者发生由旁路信号激活或表型转化等ALK非依赖性耐药机制。MET扩增是ALK-TKIs类药物重要的非依赖性耐药机制之一，约15%的ALK抑制剂复发患者中检测到MET扩增^[10]^。克唑替尼是有效的MET抑制剂，此前一项I-II期研究^[11]^发现，联合应用EGFR和MET抑制剂可诱导EGFR抑制剂耐药伴MET扩增肿瘤细胞的重新应答，但迄今为止在ALK融合阳性患者中这种联合用药的治疗经验仍然十分有限。Dagogo-Jack等^[10]^报道，在2例MET扩增的耐药患者中，ALK抑制剂（洛拉替尼）与MET抑制剂（克唑替尼）联合用药在初期治疗时取得了可观的临床疗效，但3个月后发生PD，经活组织检查提示耐药细胞可能发生了其他改变，降低了对双重ALK/MET联合治疗的敏感性。这些提示MET抑制剂联合用药在临床上是可行有效的。考虑患者化疗后发生严重骨髓抑制，PS评分较差（3分），我们首先尝试应用克唑替尼250 mg bid口服，服药后症状和胸腔积液呈好转趋势，但1个月后出现头晕症状，头部强化MRI发现发生脑转移灶，考虑克唑替尼药代动力学耐药有关，患者PS评分差，用药方案调整为克唑替尼250 mg qd口服，联合洛拉替尼50 mg qd口服，患者临床症状较前明显好转，肿瘤标志物较前下降。但遗憾的是，患者于2024年9月4日因呛咳或误吸诱发与肿瘤进展无关的肺感染，引起呼吸衰竭，导致死亡。

值得一提的是，本例ALK融合阳性肺腺癌患者同时伴有PD-L1高表达。尽管近年来免疫检查点抑制剂（immune checkpoint inhibitors, ICIs）广泛应用于缺乏靶向驱动基因阳性的NSCLC患者，但是否将其纳入ALK融合阳性伴PD-L1高表达NSCLC患者的治疗仍存在争议。PD-L1表达可通过诱导ALK融合阳性肿瘤细胞的致癌信号上调，导致PD-L1表达水平和ICIs获益之间的预测相关性丧失，从而否定了PD-L1水平作为该人群中生物标志物的可靠性^[1]^。Mazieres等^[12]^对271例KRAS基因突变、125例EGFR基因突变、43例BRAF基因突变、36例MET基因突变、29例HER2基因突变、23例ALK基因融合阳性、16例RET基因融合阳性、7例ROS1基因融合阳性以及1例复合驱动基因阳性的晚期NSCLC患者接受程序性细胞死亡受体1（programmed cell death 1, PD-1）/PD-L1抑制剂的疗效进行回顾性分析，这些患者主要在二线治疗（42%）、三线治疗（26%）或后期治疗（27%）中接受了ICIs治疗，研究结果表明PD-1/PD-L1抑制剂对EGFR、ALK和HER2亚组患者疗效有限。总的来说，现有数据并不支持ALK融合阳性伴PD-L1高表达NSCLC患者在靶向治疗进展后中接受ICIs单药治疗。然而，对于类似于本报道中靶向基因突变伴PD-L1高表达的NSCLC患者，在后线ALK-TKIs治疗失败后可尝试与化疗、免疫治疗或抗血管生成药物联合用药的治疗模式，与单独化疗或联合抗血管生成药物相比，可能会改善预后。

该病例提示基因检测对于肺腺癌患者的后线诊疗具有重要的指导意义。洛拉替尼对EML4-ALK融合V3亚型是一种有效的治疗选择，在转化为EML4-ALK融合V3亚型伴MET扩增肺腺癌后，洛拉替尼与克唑替尼或其他MET抑制剂联合用药可能会改善患者的生存情况，单药ALK-TKIs序贯治疗可能不是ALK融合阳性的转移性NSCLC患者在靶向治疗进展后逆转耐药的最佳答案，因此在后线治疗中如何联合用药来预防和克服耐药性仍需进一步探索。
