# 更正声明

**DOI:** 10.3779/j.issn.1009-3419.2025.103.01

**Published:** 2025-03-20

本刊2024年第27卷第12期刊登的题为“克唑替尼治疗洛拉替尼耐药伴*MET*扩增的*EML4-ALK*基因融合阳性晚期肺腺癌1例”［王欣怡, 穆宁, 刘梅, 等. 克唑替尼治疗洛拉替尼耐药伴*MET*扩增的*EML4-ALK*基因融合阳性晚期肺腺癌1例. 中国肺癌杂志, 2024, 27(12): 956-960.］一文中：第956页正文第1段，因个人疏忽将基因名称书写错误，现作者要求修改，将“间质-上皮细胞转化因子（mesenchymal-epithelial transition factor, *MET*）”更正为”间变性淋巴瘤激酶（anaplastic lymphoma kinase, *ALK*）”，对此深表歉意。

Erratum: Crizotinib Treatment for Lorlatinib-resistant *MET*-amplified *EML4-ALK*-fusion Positive Advanced Lung Adenocarcinoma: A Case Report

WANG X, MU N, LIU M, *et al*.

In the version of this article initially published, error appeared in the beginning of the first paragraph on page 956. Due to the authors’ personal oversight, the gene name was written incorrectly. The authors now request to correct "mesenchymal-epithelial transition factor (*MET*) 'to' anaplastic lymphoma kinase (*ALK*)."

